# Monkeypox: The Re-emerging Terror

**DOI:** 10.7759/cureus.28597

**Published:** 2022-08-30

**Authors:** Anuja Sapkal, Sachin Agrawal

**Affiliations:** 1 Medicine, Jawaharlal Nehru Medical College, Datta Meghe Institute of Medical Sciences, Wardha, IND; 2 Internal Medicine, Jawaharlal Nehru Medical College, Datta Meghe Institute of Medical Sciences, Wardha, IND

**Keywords:** virus, varicella, smallpox, orthopoxvirus, monkeypox

## Abstract

Monkeypox is a zoonotic Orthopoxvirus called human Monkeypox. It has symptoms that resemble or are pretty similar to smallpox. Monkeypox virus belongs to the genus Orthopoxvirus, which also includes cowpox, vaccinia, and variola viruses. The World Health Organization confirmed in 1970 that the primary virus is the Orthopoxvirus infecting humans after smallpox elimination. Clinically distinguishing the condition from varicella and smallpox is challenging for a clinician. Although the mortality rate of this disease is low, new tests are being tried and studied, which are required for a more accurate and quick diagnosis because the lab diagnosis is the key to the detection of illness and its monitoring. The illness or the virus is endemic to parts of western and central Africa. Surveillance in underdeveloped rural regions is challenging but manageable with evidence-based techniques and training materials for public health professionals. However, as in the present scenario, the disease is having a worldwide outbreak in various countries, and recently India detected its first case on 15 July 2022 in New Delhi. The widespread disease is due to trading exotic pets and international travel. Since smallpox vaccinations are not administered to people regularly, epidemiological studies are required. New medications and vaccines provide hope for treating and preventing Monkeypox; however, further study is required before they can be used effectively. Also, there is a requirement for advanced scientific studies in the etiology, epidemiology, and biological structure of the virus in the endemic zones to know and halt the spread of infection to humans.

## Introduction and background

Monkeypox belongs to the family: Poxviridae and species: Monkeypox virus. It is a type of zoonotic disease which means that it spreads from animals to humans. The spread of the virus can take place via various media. Monkeypox virus was first isolated when monkeys sent from Singapore to a research center in Denmark became ill and were identified in 1958 [1]. However, the virus was recovered from a toddler from Congo who was thought to be infected with smallpox in 1970, marking the first known human instance [2]. The disease also has infected squirrels, rats, mice, monkeys, and humans [3]. The virus can be transmitted from person to person too. 

The current ongoing outbreak of the Monkeypox viral disease was first confirmed on 6 May 2022. The primary group of the cases was found in the United Kingdom, where the first outbreak case was identified with a traveling history linked to Nigeria, where the disease is already endemic. The recent outbreak of the monkeypox virus among humans occurred mainly in bisexual men or gays. Therefore, male sex transmission is a significant risk factor for the spread of the infection. Henceforth, it is assumed that the monkeypox virus can occur via sexual contact and seminal fluids, including oral or non-penetrative contact. Therefore, having multiple sexual partners can also increase the overall risk of infection. 

The size of the monkeypox virus under electron microscopy is substantial (less than 250 nm). Viruses are brick-shaped and have a linear ds DNA enclosed in a lipoproteinecious sheath [4]. Poxviruses have all the required mechanisms of replicating, transcription, assembling, and special proteins in their genomic sequence, in addition to relying on the host's ribosomes for mRNA translation [5].

Epidemiological variants of Virus 

There are two different epidemiological variants of monkeypox viruses: those in West Africa and those in Central Africa. On a general note, West African variant infections show a low severity disease in humans and other primates [6]. A group of potential genes that may play a role in the varying virulence of clades were identified through comparisons between different genomes of strains from West and Central Africa. These open reading frames may affect the virus's life cycle, range of host, attack on immunity, or operate as a factor of virulence, according to predictions [7]. 

Human-generated cells from previously infected monkeypox patients cannot produce inflammatory cytokines because central African Monkeypox blocks T-cell receptor-mediated T-cell activation. According to these findings, Monkeypox might create a modulator that inhibits host T-cell responses [8]. The Central African monkeypox virus has several immune evasion options [7]. It has been suggested that a significant immune-modulating component causing the enhanced virulence of Central African strains is the complement enzymes inhibitors. This gene inhibits complement enzymes lacking in West African strains [9,10]. 

In addition, compared to West African strains of Monkeypox, Central African strains selectively suppress host responses, including apoptosis in the host body. The observed differences in pathogenicity could be caused by several loci [7,10]. Additionally, transcriptome analyses have demonstrated that during an infection, Central African Monkeypox seems to selectively mute the transcription process of genes implicated in host immunity [11]. 

## Review

Pathophysiology 

The virus multiplies at the site of the inoculation after virus entry by any media (oropharynx, nasopharynx, or subcutaneous), then spreads to nearby lymph nodes. Salivary secretions, oronasal droplets, or direct contact with exudate of the lesion or crust material are thought to be the main routes of transmission [12,13]. Another potential form of exposure is viral shedding through feces [12]. 

Evidence suggests that the members of the house or health care providers who provide care for a monkeypox patient are at high risk for contracting an illness, even though the man-to-man spread of the virus is less than smallpox. However, the rate of man-to-man transmission occurs up to 11.7% of house members of the cases who had not previously received a vaccine for smallpox [13]. The spread of the infection also occurs via close physical contact with the infected person through seminal fluid, etc. The primary viral infection then triggers the spread of the virus, and organs may act as a reservoir of the virus. This is the stage of incubation, which can last up to 21 days and usually lasts 7 to 14 days. 

The onset of symptoms is related to a secondary viral infection causing 1 to 2 days of prodromal symptoms. The symptoms can be fever and lymphadenopathy. Symptoms precede the lesions, and the infected patients can remain highly infectious. Lesions begin in the oro-pharyngeal region and then appear on the skin. When lesions appear, serum antibodies are often detected [14]. 

Clinical features

The incubation period of Monkeypox ranges from five days to three weeks [15,16], and symptoms can last for nearly 2 to 5 weeks. The Monkeypox infection is divided into three stages: incubation, prodrome, and rash. Fever, headache, myalgia, weakness, and lymphadenopathy are among the early signs of Monkeypox, which sets it apart from smallpox. After one to two days, lesions on the face, limbs, soles, palms, and mucosal lesions of the mouth appear. These lesions are centrifugally concentrated. The approximate number of lesions may vary from a few to thousands. The rash may or may not spread to the rest of the body parts [17]. 

The lesions progress at 1- to 2-day intervals through macule, papule, vesicle, and pustule phases throughout the ensuing 2 to 4 weeks. Lesions range in size from 2 to 10 mm, are hard, and undergo synchronous change. Before crusts form, lesions are in the phase of pustules for almost a week or seven days. Most often, the illness clears itself 3 to 4 weeks after the commencement of symptoms, with crusts developing and desquamating during the following 7 to 14 days. Patients are no longer considered to be infective once all crusts fall off. After healing, the lesion may leave pale marks before turning into dark scars [18]. 

Monkeypox is a self-limited disease. The severity of the disease is related to the period of contact with the virus and the patient's immune status. In the recent 2022 outbreak, many cases of the monkeypox outbreak presented with genital and peri-anal lesions, fever, swollen lymph nodes, and pain when swallowing, with some patients manifesting only single sores from the disease. 

**Figure 1:**
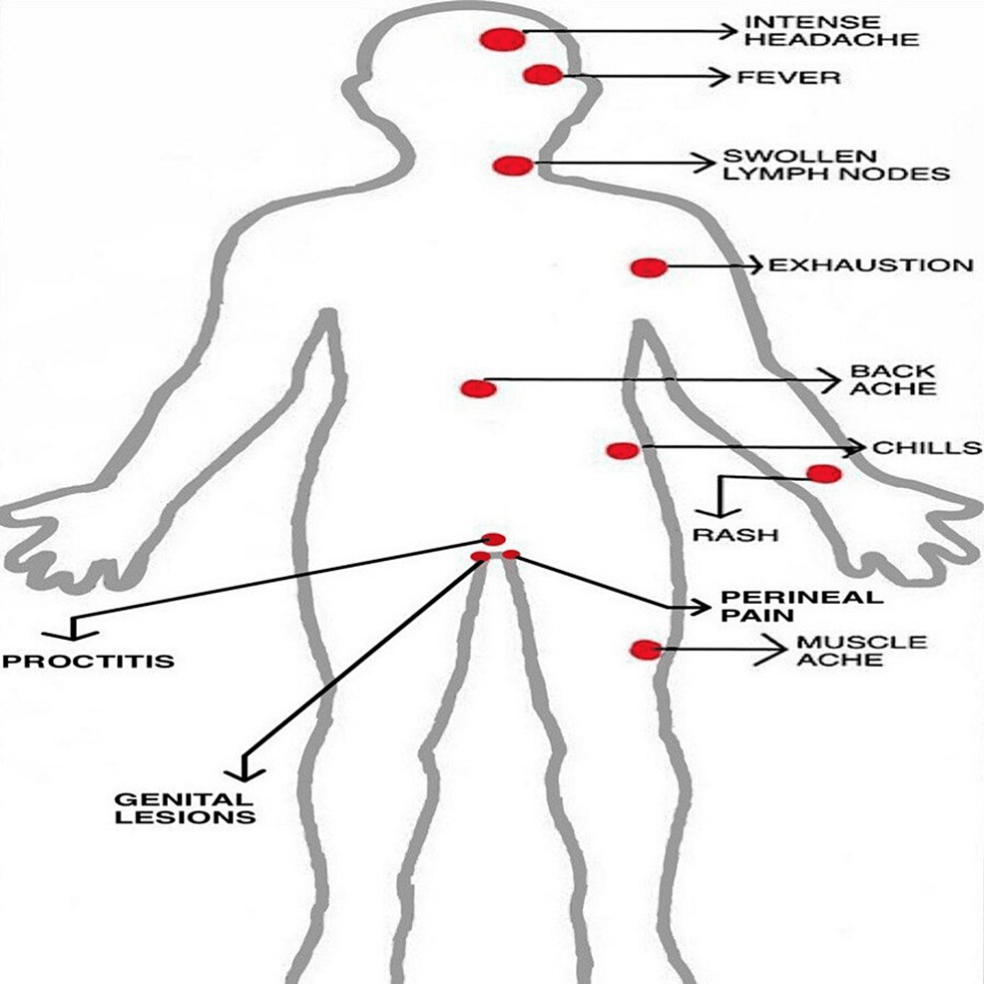
Showing symptoms of Monkeypox Virus

In a study, the involvement of the anorectal mucosa was reported as the presenting symptom in 61 persons; this involvement was associated with anorectal pain, proctitis, tenesmus, or diarrhea (or a combination of these symptoms) as mentioned in the following figure 1. Oropharyngeal symptoms were reported as the initial symptoms in 26 persons, including pharyngitis, odynophagia, epiglottitis, and oral or tonsillar lesions [19]. 

Complication

Unvaccinated patients (74%) had more severe problems and sequelae than patients who had received vaccinations (39.5%). Indicative of a secondary lung infection, patients are also seen with respiratory discomfort or bronchopneumonia, a frequent late manifestation of the disease. By the end of the second week of the illness, vomiting or diarrhea can start, which may significantly worsen the dehydration. One patient had encephalitis, while another with more than 4500 lesions had septicemia [20]. Ocular infections are a possibility, and they can cause permanent cornea scarring and irreversible loss of vision [21]. 

The widespread, long-term consequence of infection survivorship is pitted scarring. Unvaccinated patients have an average case-fatality rate of up to 11% and can lead to death [22], and youngsters are frequently more susceptible to severe disease types. Other complications include hyperpigmentation or hypopigmentation of the skin, keratitis, pneumonitis, encephalitis, and bacterial superinfection-easy access to medical facilities, improved testing methods, and efficiency. The construction of healthcare setups restricts the capability of making the best decisions about dealing with this ignored tropical disease [23]. Hence, there is a scope for improvement that can make this disease easily treatable and eradicate it in the future. 

Diagnosis

The diagnostic assay remains the most critical part of identifying the Orthopoxvirus. Conventional tests, including virus isolation from a clinical specimen, electron microscopy, and IHCs, are still reliable, but they need complex lab equipment and highly skilled technicians.

Real-time polymerase chain reaction (PCR) specimen analysis can determine whether Monkeypox or Orthopoxvirus is present in a lesion sample [24-27]. All these tests are susceptible and effective at finding the DNA of the virus. Since real-time PCR is now most effective in large lab settings, its application as a real-time diagnostic in rural or village areas and resource-limited settings is still limited. Real-time PCR for diagnostic purposes may become more practical outside large laboratories due to technological advancements. 

Antibody-based diagnostics are required to identify the root cause of instances discovered retroactively. Orthopoxvirus cross-reactivity makes anti-Orthopoxvirus immunological assays effective in situations when the virus that is causing sickness has already been identified. For the individuals who came in contact with an orthopoxvirus, including by vaccination, anti-Orthopoxvirus immunoglobulin G (IgG) alone cannot offer a conclusive diagnosis. As an alternative, serological assays which measure anti-Orthopoxvirus immunoglobulin M (IgM) are better suited to identify acute retrograde illness, even in people who have previously had vaccinations [28]. 

Utilizing lesion samples from acute orthopoxvirus infections, a recent Tetracore Orthopox BioThreat Alert pilot produced encouraging results. In preparations with 107 plaque-forming units/mL, this technique consistently recognized the vaccinia and monkeypox viruses, and 5 out of 6 evaluated clinical specimens were correctly identified [29]. 

Cases of the monkeypox virus frequently want diagnosis and treatment at outlying clinics or healthcare setups devoid of electricity; as a result, there is a mandatory need for the creation of assays that can be used to conduct testing in rudimentary settings with no staff training. 

The various differential diagnosis of the Monkeypox Virus can be as follows: Smallpox; Chickenpox; Generalized vaccinia; Disseminated zoster; Disseminated herpes simplex; Syphilis; Rickettsial pox; Measles; Drug-associated eruption (table 1) [17].

**Table 1:** Showing differences between Monkeypox, Smallpox, Varicella

Features	Monkeypox	Smallpox	Varicella
Period of incubation	One-Two weeks	One-Two weeks	One-Three weeks
Fever	+(38.5-40.5 deg Celsius)	+(above 40 deg Celsius )	+(upto 38.8 deg Celsius)
Malaise	+	+	+
Headache	+	+	+
Lymphadenopathy	+	-	-
Lesion pattern	centrifugal pattern	centrifugal pattern	centripetal pattern
Appearances of lesion	Deep seated and hard,well circumscribed.	Deep seated and hard ,well circumscribed.	Superficial,irregular, dew drop appearance

Therapy and vaccinations

Various compounds have shown promising action against the monkeypox virus with the advancement in therapeutics. Cidofovir, an antiviral agent, acts efficiently against various viruses by blocking viral DNA polymerase. 

The nephrotoxicity associated with cidofovir has been reduced in the modified cidofovir molecule known as CMX-001. Numerous orthopoxvirus species have been used to demonstrate the antiviral activity of CMX-001. The medication ST-246 has demonstrated good efficacy against several orthopoxvirus species, including the variola virus. It prevents the release of the virus from the cell (intracellularly). 

These substances have been utilized in various combinations to treat severe vaccine-related adverse effects, including with vaccinia immune globulin [30]. In a few patients, vaccination after disease exposure is recommended with modified vaccinia, Ankara vaccine (smallpox and monkeypox vaccine, live, non-replicating). This vaccine does not cause a skin rash and increases the chance of localized or widespread dissemination [17]. 

Additionally, clinical studies have demonstrated that modified vaccinia Ankara is safe and promotes the formation of antibodies in individuals with atopy and weakened or low immunity status -conditions that are said to be contraindicated for the injection of live vaccinia [31]. The CDC states that immunization within four days of exposure may stop the start of the disease, and immunization within 14 days may lessen its severity.

Disinfectants and biocidal agents used against Monkeypox

In suspension experiments, 70% ethanol (for 1 minute), 0.2% peracetic acid (for 10 minutes), and 1-10% of a probiotic cleanser (for 1 hour) could cause all inactive vaccine viruses by at least four log10, which was most frequently demonstrated with various types of organic load. In suspension experiments, hydrogen peroxide (14.4%) and iodine (0.04-1%) worked well. Artificially polluted surfaces responded well to sodium hypochlorite (0.25-2.5%) and 2% glutaraldehyde (10 min). In 3 minutes, copper (99.9%) was equally efficient against the monkeypox and vaccinia viruses. 

Evolution Of Poxvirus 

The evolution of zoonotic infections to become more contagious or virulent in humans is a big worry. One concern with orthopoxviruses like monkeypox virus is the likelihood that they could develop into diseases that could cause another pandemic similar to smallpox. As a viral DNA polymerase enzyme replicates the DNA genome with 3'-5' exonuclease proofreading activity, xenovirus has a lower mutation rate than RNA viruses [32]. 

According to molecular clock studies, poxviruses have substitution rates ranging from 2 × 10−6-1 × 10−5 substitutions of nucleotides per site each year [33], which might lead to as many modifications of 2 nucleotides in the genome annually. It is thought that during early poxvirus evolution, gene duplication and subsequent diversification expanded the number of auxiliary gene families [34]. At least in lab settings, gene multiplication also seems to be a fairly typical way for poxvirus to adjust to the environment quickly. Drug-resistant VACV mutants, such as those resistant to rifampin or hydroxyurea, which inhibit several phases of VACV replication, developed very quickly when these medicines were added during passage [35,36]. 

 Many of the mutants have tandem repeats of a viral gene in their genomes that is either the direct drug target or a near companion of the drug target [35,36], enabling them to reduce the medication effect by boosting the gene dosage. Similar to how VACV rapidly extended their genome by copying a viral gene (K3L or a related gene) that encodes a weak human PKR inhibitor, VACV did the same to circumvent the activity of the human antiviral protein PKR [37,38]. After the gene was amplified, a helpful point mutation appeared in one of the K3L gene copies, which then permitted the contraction of the K3L tandem repeats into the one with the point mutation [37]. 

Gene accordions, a multi-step evolutionary process, may hasten the evolution of poxviruses [37]. Recombination allows the pox virus to accommodate the insertion of substantial amounts of foreign DNA into their genomes, making VACV an effective vaccine delivery vehicle. Poxviruses can recombine highly in a lab setting, and certain naturally occurring poxvirus recombinants have been discovered. The mosaic genome of a novel Chickenpox virus strain discovered in a Norwegian patient may have resulted through recombination with three different variants of the Orthopoxvirus species [38]. Many poxvirus variants, including two unique OPXV species identified from human patients in this century, have signs of recombination with OPXV [39]. 

In order to prevent the Monkeypox infection and the spread of the disease following steps are to be followed: Stay at home and avoid contact with the infected person or those with symptoms; Practice safe sex, avoid multiple sexual partners, and avoid close physical contact with anonymous; Use masks to maintain good hand hygiene and respiratory practices, like covering your mouth with a handkerchief or tissue while coughing and sneezing; Avoid touching contaminated surfaces; clean and disinfect them; Use a personal protective kit if caring for an infected person; Eat thoroughly cooked food items that contain animal meat or parts.

## Conclusions

The spread of Monkeypox has been endemically seen in more than 20 countries since May 2022. The degree of infectivity of Monkeypox is relatively less, and its chances of becoming a pandemic are meager. It is crucial to educate humans and healthcare professionals in areas where the monkeypox virus has already been affected or in areas that are already at high risk of exposure. The best protection against global spread is local confinement. Enhancing patient awareness of the illness, reporting accuracy, and accessibility to diagnostic tools are essential for gathering the data required to comprehend Monkeypox more thoroughly and develop a more vigorous defense against it. The civil war between the countries and forced migration raise worries about the spread of the virus into a monkeypox-free region or about people moving to more heavily forested areas where they are more likely to come into contact with wildlife and a variety of zoonoses. In order to better comprehend the variety of elements involved in the transfer of the disease and distribution, the recorded increase in the frequency of human disease requires deeper investigation and analysis. Improving our knowledge of these essential microorganisms will assist in better direct preventive tactics and lessen human disease. There are still numerous mysteries concerning human disease, animal reservoirs, and the virus. Easy access to medical facilities, improved testing methods, and it is efficiency. The construction of healthcare setups restricts the capability of making the best decisions about dealing with this ignored tropical disease. Hence, there is a scope for improvement that can make this disease easily treatable and eradicate it in the future. We should strengthen the fundamental research on zoonotic poxvirus disease and pay close attention to scientific organization. It is crucial to increase awareness and surveillance to stop the spread of Monkeypox. Early detection is crucial to stopping the monkeypox outbreak, and international technical cooperation is crucial to lowering the danger of Monkeypox.
